# Contribution of optical resolution to the spatial precision of two-photon optogenetic photostimulation *in vivo*

**DOI:** 10.1117/1.NPh.11.1.015006

**Published:** 2024-02-06

**Authors:** Robert M. Lees, Bruno Pichler, Adam M. Packer

**Affiliations:** a Science and Technology Facilities Council, Octopus Imaging Facility, Oxfordshire, United Kingdom; b University of Oxford, Department of Physiology, Anatomy, and Genetics, Oxford, United Kingdom; c Independent NeuroScience Services INSS Ltd., East Sussex, United Kingdom

**Keywords:** optogenetics, two-photon microscopy, calcium imaging, photostimulation, neurobiology, systems neuroscience, all-optical

## Abstract

**Significance:**

Two-photon optogenetics combines nonlinear excitation with noninvasive activation of neurons to enable the manipulation of neural circuits with a high degree of spatial precision. Combined with two-photon population calcium imaging, these approaches comprise a flexible platform for all-optical interrogation of neural circuits. However, a multitude of optical and biological factors dictate the exact precision of this approach *in vivo*, where it is most usefully applied.

**Aim:**

We aimed to assess how the optical point spread function (OPSF) contributes to the spatial precision of two-photon photostimulation in neurobiology.

**Approach:**

We altered the axial spread of the OPSF of the photostimulation beam using a spatial light modulator. Subsequently, calcium imaging was used to monitor the axial spatial precision of two-photon photostimulation of layer 2 neurons in the mouse neocortex.

**Results:**

We found that optical resolution is not always the limiting factor of the spatial precision of two-photon optogenetic photostimulation and, by doing so, reveal the key factors that must be improved to achieve maximal precision.

**Conclusions:**

Our results enable future work to focus on the optimal factors by providing key insight from controlled experiments in a manner not previously reported. This research can be applied to advance the state-of-the-art of all-optical interrogation, extending the toolkit for neuroscience research to achieve spatiotemporal precision at the crucial levels in which neural circuits operate.

## 1 Introduction

Interrogating neural circuits requires observation and intervention at the spatiotemporal scales that these circuits operate, i.e., spatially at the cellular level (∼10  μm) and temporally within milliseconds. Two-photon optogenetics^1^^–^^6^ refers to the use of two-photon excitation to photostimulate opsin proteins embedded in the cellular membranes to activate or inhibit cells in a targeted fashion. The use of two-photon excitation enables targeting at the scale of individual neurons deep in scattering tissue, for example, up to ∼0.5  mm deep in the brains of awake, behaving rodents. This approach extends the optogenetic toolkit beyond bulk activation using fiber-coupled LEDs by enabling researchers to target cells with greater specificity. For example, neurons can be targeted based on their functional properties instead of, or in addition to, genetic or viral targeting approaches. Combined with holography for multiplexed targeting and calcium imaging for neural activity recording, two-photon optogenetics becomes a versatile all-optical interrogation^7^^–^^9^ approach for reading and writing neural activity *in vivo* with unprecedented precision.

Since the beginning of two-photon optogenetics, a key goal has been to target neurons for activation with single-cell precision. One of the key metrics for quantifying single-cell precision is the physiological point spread function (PPSF), which was defined by Pégard et al.^10^ as “the photocurrent response to multiphoton photostimulation as a function of the displacement between the holographic target and the patched cell.” First, a recording of neural activity from a single neuron is established either by patch-clamp electrophysiology, the gold standard for recording subthreshold depolarisation and/or spiking activity, or by calcium imaging, a widely used indirect recording technique for spiking activity. Next, the photostimulation light is directed to the neuron to elicit a measurable response (i.e., photocurrent, action potential, or calcium transient). Finally, the light is moved away from the cell in a stepwise fashion laterally and axially to determine the neural responses as a function of distance between the light and the targeted neuron. One key summary metric from such recordings is the full-width at half-maximum (FWHM) of the response versus the axial dimension because the native resolution of two-photon excitation produced at the focus of an imaging system is generally worse in the axial dimension than the lateral dimension. The best case scenario would be that the axial FWHM of the PPSF is approximately equal to the diameter of the neuron being targeted, such that if the light is half of one cell’s diameter away from that cell, the activation of that neuron drops to half of its maximal value (assuming that opsin is spread uniformly around the cell body). This optimum may not be achieved if the cell is not a perfect sphere, which cells rarely are when in a native tissue environment.

The factors that affect the PPSF can be separated into three categories: optical, optogenetic, and cellular. The optical factors are the properties of the optical point spread function (OPSF) that delivers light to the sample, how much laser power is directed at the sample, the laser exposure time, and whether the beam is scanned across the cell in a specific way such as a raster or spiral. The optogenetic factors are the number of opsin proteins activated, the conductance of each, and their locations in the cell. The cellular factors are the size and shape of the cell and the depolarization required to reach action potential threshold. The action potential threshold depends on the cell’s intrinsic electrophysiological properties, such as resting membrane potential, input resistance, and ion channel expression. There are, of course, other factors that affect such experiments, such as the wavelength of light used and the number of targets that can be simultaneously addressed, but these factors do not directly affect the PPSF beyond the key factors described above.

Researchers have attempted to optimize each of these categories of factors. Regarding the optical factors, researchers have employed advanced light shaping approaches, such as temporal focusing.^11^^,^^12^ Regarding the optogenetics factors, researchers have developed stronger opsins^13^^–^^15^ and employed targeted expression strategies, such as perisomatic restriction.^16^^–^^18^ Regarding the cellular factors, researchers have tailored the activation to each cell^19^^–^^21^ to optimally reach that cell’s threshold.

Many papers have reported both optical PSF and physiological PSF results under a wide range of optical and biological conditions (see Table 1). In general, the PPSFs are larger than the OPSFs and larger than the desired outcome, i.e., they are larger than the size of the neurons being targeted. Mouse neurons are on the order of 10 to 20  μm in diameter, but the PPSFs are generally in the 20 to 40  μm range, with recent papers employing perisomatic restriction *in vivo* all hovering around 30  μm despite using different optical targeting approaches and opsins. Zebrafish neurons are smaller, generally <10  μm, but again the reported PPSFs from papers targeting these neurons are 10 to 15  μm.

**Table 1:** Physiological PPSFs from published papers^2^^,^^3^^,^^5^^–^^8^^,^^10^^,^^13^^,^^14^^,^^18^^,^^22^^–^^32^ using two-photon optogenetic excitation of neurons. The lowest axial FWHM values tend to come from papers using perisomatic restriction and/or temporal focusing. Note the best consistent results from zebrafish neurons at 10 and 15  μm.

Author	Year	Slice or *in vivo*	Type of cell	Opsin	Perisomatic restriction?	Measurement	Method	Axial FWHM (μm)
Rickgauer^3^	2009	Slice	HEK293T	ChR2	No	Photocurrent	Spiral	∼30
Papagiakoumou^22^	2010	Slice	Mouse pyr.	ChR2	No	Photocurrent	TEFO	26
Andrasfalvy^2^	2010	Slice	Rat & mouse pyr.	ChR2	No	Photocurrent	TEFO	∼30
Packer^5^	2012	Slice	Mouse pyr.	C1V1(T)	No	Spiking	Raster	30
Prakash^6^	2012	Slice	Mouse pyr.	C1V1(T)	No	Spiking	Raster	13
Rickgauer^7^	2014	*In vivo*	Mouse CA1	C1V1(TT)	No	Optical	TEFO	20
Packer^8^	2015	*In vivo*	Mouse pyr.	C1V1(T)	No	Spiking	Spiral	47
Baker^18^	2016	Slice	Mouse pyr.	ChR2-Kv2.1	Yes	Spiking	TEFO	23
Carrillo-Reid^23^	2016	*In vivo*	Mouse pyr.	C1V1	No	Optical	Spiral	∼30
Dal Maschio^24^	2017	*In vivo*	Zebrafish	ChR2	No	Optical	CGH	10
Shemesh^25^	2017	Slice	Mouse V1	soCoChR	Yes	Photocurrent	CGH	43
Shemesh^25^	2017	Slice	Mouse V1	soCoChR	Yes	Photocurrent	TEFO	24
Pegard^10^	2017	Slice	Mouse pyr.	ST-Chronos or ST-ChrimsonR	Yes	Spiking	3D-SHOT	28
Pegard^10^	2017	*In vivo*	Mouse pyr.	ST-Chronos or ST-ChrimsonR	Yes	Spiking	3D-SHOT	29
Pegard^10^	2017	*In vivo*	Mouse pyr.	ST-ChrimsonR	Yes	Spiking	3D-SHOT	29
Mardinly^13^	2018	*In vivo*	Mouse pyr.	ST-ChroME	Yes	Spiking	3D-SHOT	28
Forli^26^	2018	*In vivo*	Mouse pyr.	ChR2-Kv2.1	Yes	Spiking	CGH	34
Yang^28^	2018	*In vivo*	Mouse pyr.	C1V1	No	Spiking	Spiral	∼40
Yang^28^	2018	*In vivo*	Mouse pyr.	C1V1	No	Optical	Spiral	∼40
Marshel^14^	2019	*In vivo*	Mouse pyr.	ChRmine-Kv2.1	Yes	Optical	Spiral	26
Marshel^14^	2019	*In vivo*	Mouse pyr.	ChRmine-Kv2.1	Yes	Optical	Spiral	28
Gill^29^	2020	*In vivo*	Mouse mitral & granule	ChrimsonR	No	Optical	CGH	60
Dalgleish^31^	2020	*In vivo*	Mouse pyr.	C1V1-Kv2.1	Yes	Optical	Spiral	40
McRaven^30^	2020	*In vivo*	Zebrafish	CoChR-Kv2.1	Yes	Spiking	CGH	15
Daie^32^	2021	*In vivo*	Mouse pyr.	ST-ChrimsonR	Yes	Optical	Spiral	80
Forli^27^	2021	*In vivo*	Mouse pyr.	stCoChR	Yes	Spiking	CGH	42
Bounds^20^	2023	Slice	Mouse pyr.	ST-ChroME	Yes	Spiking	3D-SHOT	28
Oldenburg^21^	2024	*In vivo*	Mouse pyr.	ST-ChroME or ChroME2s	Yes	Optical	3D-SHOT	37
Jin^33^	2022	*In vivo*	Mouse pyr.	ChRmine-Kv2.1	Yes	Optical	CGH (beaded ring)	26

It is unclear which factors can be further optimized to match the PPSF to the approximate size of the neuron being targeted. It is also unclear which factors are already nearly optimal under the variety of experimental conditions used. We sought to answer one specific aspect of these general queries: how much optical resolution is necessary? We filled this gap in knowledge by performing a controlled experiment varying only the optical resolution while holding all other factors constant. We found there is no effect when halving the FWHM of the OPSF (from 19 to 8  μm). We hypothesize that this is primarily because the cell size and shape determine the PPSF under these conditions. Additionally, we are likely working in a condition in which out-of-focus excitation is occurring.

## 2 Materials and Methods

### 2.1 Animals, Viruses, and Surgery

All animal experimentation was carried out with approval from the UK Home Office and University of Oxford Animal Welfare and Ethical Review Board. Three wildtype (C57Bl/6) mice and three mice transgenically labeled with GCaMP6s in excitatory neurons [B6;DBA-Tg(tetO-GCaMP6s)2Niell/J x CamK2a-tTa(AI94)] were used in this study.

Briefly, a cranial window, consisting of two #1 thickness cover glasses (3 and 4 mm diameter) adhered to one another using UV-curing optical adhesive, was implanted over the somatosensory cortex (−1.9  mm anterior and +3.8  mm lateral from Bregma) of each mouse during anaesthetized recovery surgery. During surgery, ∼800  nl of C1V1-Kv2.1 virus (AAV2/9-CaMKIIa-C1V1-t/t-kv2.1-mScarlet; $∼2\times 10^{12}\;\mathrm{GC}/\mathrm{ml}$ diluted 1:5) was injected at 300  μm deep below the pial surface. In the case of wildtype mice, GCaMP6s virus (AAV2/1-syn-GCaMP6s-WPRE-SV40; $∼2.5\times 10^{13}\;\mathrm{GC}/\mathrm{ml}$ diluted 1:10 in sterile phosphate-buffered saline) was also injected at the same time. The animals were allowed to recover for at least 3 weeks before imaging, which also allowed enough time for the virus to express in neurons.

### 2.2 Microscopes and Lasers

The microscope used in this study was a Scientifica HoloStim3D (from Scientifica, United Kingdom), equipped with a fixed wavelength 920 nm laser for imaging (Axon 920 from Coherent Inc. or FemtoFiber Ultra 920 from Toptica Photonics) and a 1030 nm fixed wavelength laser for photostimulation (Satsuma ${\mathrm{HP}}^{2}$ from Amplitude Laser), each with their own dedicated scan path. The HoloStim3D was equipped with a spatial light modulator (SLM; 1920×1152 calibrated for 1064 nm from Meadowlark Optics), which allowed for deflection of the photostimulation beam axially to the imaging plane without affecting the z position of the imaging plane in the sample. ScanImage acquisition software (MBF Bioscience, Williston, Vermont, United States), written in MATLAB, was used to control all imaging and photostimulation parameters. The microscope was equipped with a 16× magnification objective (Nikon 16×, 0.8 NA, N16XLWD-PF from Thorlabs), which was used throughout all calibrations, measurements, and experiments.

### 2.3 Varying and Measuring Optical Point Spread Function

The effective numerical aperture of the photostimulation beam path was altered by cropping the 1920  pixels×1152  pixels phase mask loaded onto the SLM. The undeflected zeroth order beam at the center of the photostimulation field of view (FOV) was blocked with an optical flat and a small piece of metal, so a single-spot hologram could be measured from the first order deflections. The PSF of the photostimulation beam was estimated by imaging 0.2  μm beads (TetraSpeck Microspheres T7280 from ThermoFisher Scientific) that were dried onto a microscope slide using ethanol. Images for PSF estimation were acquired at 16× magnification with 256×256  pixels, 0.1  μm pixel size, 1  μm between slices in the axial dimension, and 5 mW average power. Individual beads that were bright and did not get damaged by the imaging process were cropped and chosen for PSF estimation. The resultant stacks were registered using the FIJI^34^ plugin StackReg^35^ to remove any tilt in the PSF. The MetroloJ^36^ plugin for ImageJ was used to fit a Gaussian curve to each individual bead in the axial and lateral dimensions. The curve was manually inspected for a good fit between the model and the data. At least three beads were imaged for each PSF to ensure that results were consistent.

### 2.4 Photostimulation

To determine whether fast versus slow spiraling was better, fast spiraling was done using 25×10  ms spirals, whereas slow spiraling used 1×250  ms spiral. For the comparison, each neuron was measured with both fast and slow spiralings, in a random order. Slow spiraling was used for all experiments. 6 mW average laser power was used for photostimulation in these experiments.

Subsequently, to find the best photostimulation laser power, different neurons were targeted for each power of 2, 3, 4, and 5 mW average power, and no neuron was repeated with multiple laser powers.

For all further experiments, the 1030 nm photostimulation laser was used at 3 mW average power as measured by a sensor (S130C from Thorlabs) at the objective. The power was measured separately for each axial position and for each effective numerical aperture adjustment. The repetition rate of laser pulses was set to 2 MHz, with ∼350  fs pulse width and ∼4300  W peak power, and the energy per pulse was ∼1.5  μJ. The SLM was kept at a temperature of ∼25°C.

Each axial position for the photostimulation beam was controlled by adjusting the diffraction from the SLM to focus the hologram deeper or shallower relative to the imaging plane. The first-order deflection was ∼100  μm lateral to the zeroth order. The zeroth order was blocked at a conjugate plane with a piece of metal to reflect/absorb the beam. The exact positions were calibrated and measured by burning a spot into a plastic slide (Autofluorescent Plastic Slides from Chroma) at each depth. The spots ranged axially from −60  μm to +60  μm relative to the imaging plane in steps of 15  μm with 9 steps in total.

Using separate galvanometers from the imaging path, the single-spot hologram was spiraled with five concentric revolutions across the target neuron with a diameter of 10  μm at 3 mW average power. Repeats were carried out every 10 s to maintain photostimulation efficacy, with three repeats per neuron per axial position. The interval between each axial position was at least 30 s. Stimulations alternated between the “standard” direction from −60 to +60  μm or the “reverse” direction from +60 to −60  μm, and the optical resolution was also alternated. Each neuron received stimulation in only one direction with only one optical resolution.

### 2.5 Imaging

Responses to photostimulation were imaged at 30 Hz using resonant scanning and a wavelength of 920 nm. Images consisted of 512×512  pixels with a pixel size of ∼1.25  μm. Two separate detectors (multialkali PMTs) were used to collect emission from C1V1-Kv2.1-mScarlet and GCaMP6s. Group delay dispersion (GDD) was optimized for the imaging laser by finding the highest fluorescent signal in a uniformly fluorescent sample while adjusting the GDD. To reduce imaging-induced photoactivation of C1V1, a large imaging FOV of 650  μm square was chosen to reduce the time that the imaging beam dwelt on each neuron. An average imaging laser power of 20 mW was chosen to further reduce the effects of imaging-induced photoactivation, and this power level was determined by scanning at different imaging powers and measuring the change in GCaMP fluorescence over a 30-s period. A power of 20 mW resulted in no ramp-up of activity across the duration of imaging (data not shown).

During *in vivo* experiments, animals were head-fixed while awake, and images were acquired at 150  μm deep in the somatosensory cortex (estimated layer 2). The cranial window was flattened by tipping/tilting the animal holder so that the window was perpendicular to the objective lens, resulting in less distortion of the images from refraction.

All imaging and photostimulation was synchronized using the voltage input/output recording software, PackIO.^37^ A voltage trigger was used to begin imaging, and photostimulation was manually triggered from ScanImage. Both the frame clock and galvanometer output were recorded to detect spiral initiation times relative to imaging frames.

### 2.6 Data Analysis

The voltage recordings of the frame clock and photostimulation trial onsets were used to find the trial start times. Mean raw fluorescence trials were calculated across the three repeats for each neuron, as described in the rest of this paragraph. Imaging data from different axial photostimulation positions were aligned to each other using ImageJ StackReg plugin to ensure that there was no drift between different acquisitions in the same FOV. The normalized change in fluorescence was calculated using the ImageJ Image Calculator tool as follows: $\Delta F/F=(F-F_{0})/F_{0}$, where F is the raw fluorescence and $F_{0}$ is the mean F in the baseline period (1.5 s before the stimulation onset). A freehand region of interest was drawn around the stimulated neuron using the ImageJ freehand drawing tool, and the mean ΔF/F of all of the pixels was taken after importing the data back into Python. Control ROIs that were not photostimulated were drawn ∼100  μm away from the targeted neuron with the same shape as the neuronal ROI and analyzed in the same way. The response for each trial was taken as the mean ΔF/F of the period post-stimulation (from 0 to 1.5 s after the photostimulation ended). Any neuron that did not respond at a single-axial position with a mean of ≥0.35 ΔF/F in the 1.5 s post-photostimulation was removed as it suggested that the photostimulation was unsuccessful. This was based on the average response to stimulations in the 3 mW condition of the laser power photostimulation tests [0.37 ΔF/F versus 0.15 ΔF/F for sample versus control regions; Figs. 2(b) and 2(c)]. There is no photostimulation artifact reduction strategy to prevent fluorescence from the photostimulation laser excitation appearing in the imaging channel for the duration of the photostimulation period. Overall, 15/31 neurons were included for the large OPSF, and 17/38 neurons were included for the small one.

Due to the uncertainty of the exact position of the neurons in the axial dimension, the photostimulation precision curves were offset so that the maximum response z offset was relabeled as 0  μm. No curve was shifted by more than 15  μm (one SLM z offset position). The photostimulation precision for the different optical resolutions was measured as the FWHM (the width of a horizontal line intercepting the signal at half the prominence of the peak of the curve) using the find_peaks and peak_widths functions as part of SciPy’s signal processing module for Python. Standard (−60 to +60  μm) and reverse (+60 to −60  μm) photostimulation response curves were combined by flipping the reverse curves, so the positions matched the standard condition.

For ΔF/F timeseries plotting, a rolling average of 5 frames was used. All error bars are ±95% confidence interval, unless otherwise stated.

### 2.7 Modeling

MATLAB was used to generate mock data for an axial OPSF from the experimental results. For each OPSF, a standard deviation was calculated from the FWHM value, and a Gaussian distribution was generated from random numbers using that standard deviation. The data were normalized to the maximum values, and a moving average filter of 5 was applied. Cell sizes were modeled as binary square waves, in which the y axis was 1 across the entire cell diameter on the x axis. Each OPSF was convolved with the square wave of each cell size using the convolve function. The code and processed data are freely available to repeat these measurements and can be found at https://github.com/Packer-Lab/OPSF_vs_PPSF

## 3 Results

### 3.1 Varying the Photostimulation OPSF Axial Spread Using a Spatial Light Modulator

**Fig. 1:**
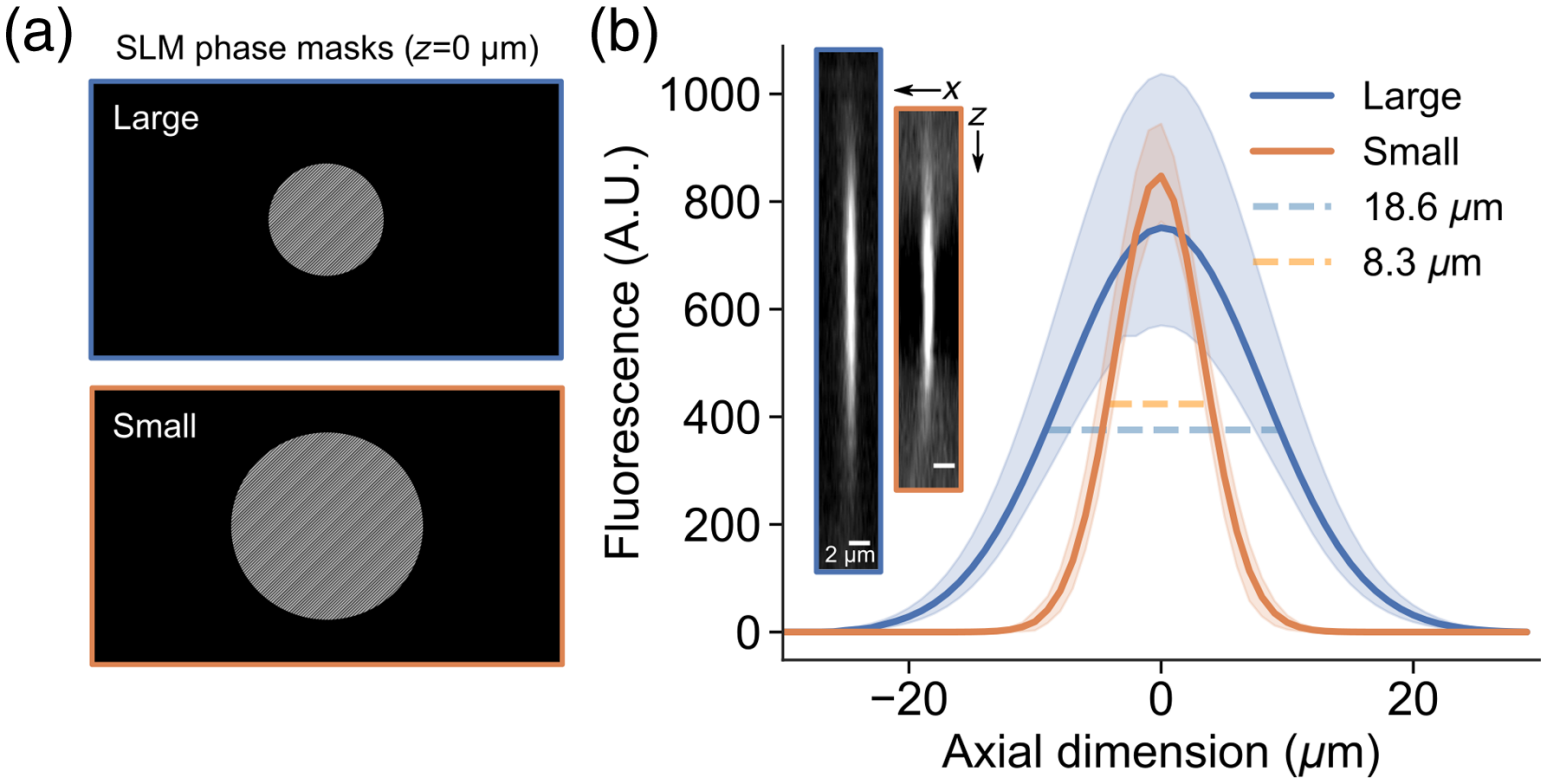
Varying the axial spread of the photostimulation OPSF using an SLM. (a) Example phase masks applied to the SLM active area. These phase masks were used to vary the axial spread of OPSFs; the examples are from the 0  μm photostimulation z position. (b) Mean Gaussian curves ±95% CI (confidence interval) fitted to the z profile of 0.2  μm fluorescent beads imaged using the photostimulation OPSF (large OPSF, n=3 beads; small OPSF, n=4 beads). The FWHM is indicated (dashed line), showing the axial extent of the OPSF. Inset is example images from a bead imaged with the “large” (blue outline) and “small” (orange outline) OPSFs. The images are xz projections; brightness and contrast have been adjusted separately for the two images to highlight the shape.

We used an SLM to vary the size of the photostimulation OPSF and control its z position relative to the targeted neuron. The phase masks being applied to the SLM active area resulted in a single focused laser spot at the sample, roughly 100  μm from the center of the photostimulation FOV. To elongate the point spread function axially, we cropped the phase masks [Fig. 1(a)], effectively underfilling the back aperture of the objective. We imaged submicron fluorescent beads using the 1030 nm photostimulation beam of the SLM path to measure the axial extent of the OPSF produced by these phase masks. The “small” OPSF had an axial FWHM of 8.3  μm, and the “large” OPSF had an FWHM of 18.6  μm [large OPSF, n=3 beads; small OPSF, n=4 beads; Fig. 1(b)]. We offset the z position of the OPSF in 15  μm steps from −60 to +60  μm relative to the imaging plane by creating a new phase mask for each position. This was confirmed by burning the resultant holograms into a fluorescent plastic slide and measuring the z position of the burns relative to the imaging plane using the motorized stage (data not shown). In summary, we created large and small OPSFs that we could position in three dimensions to target individual neurons and test the effect of axial OPSF size on photostimulation precision.

### 3.2 Optimizing Photostimulation for Detection of Nonsaturated Neuronal Responses

**Fig. 2:**
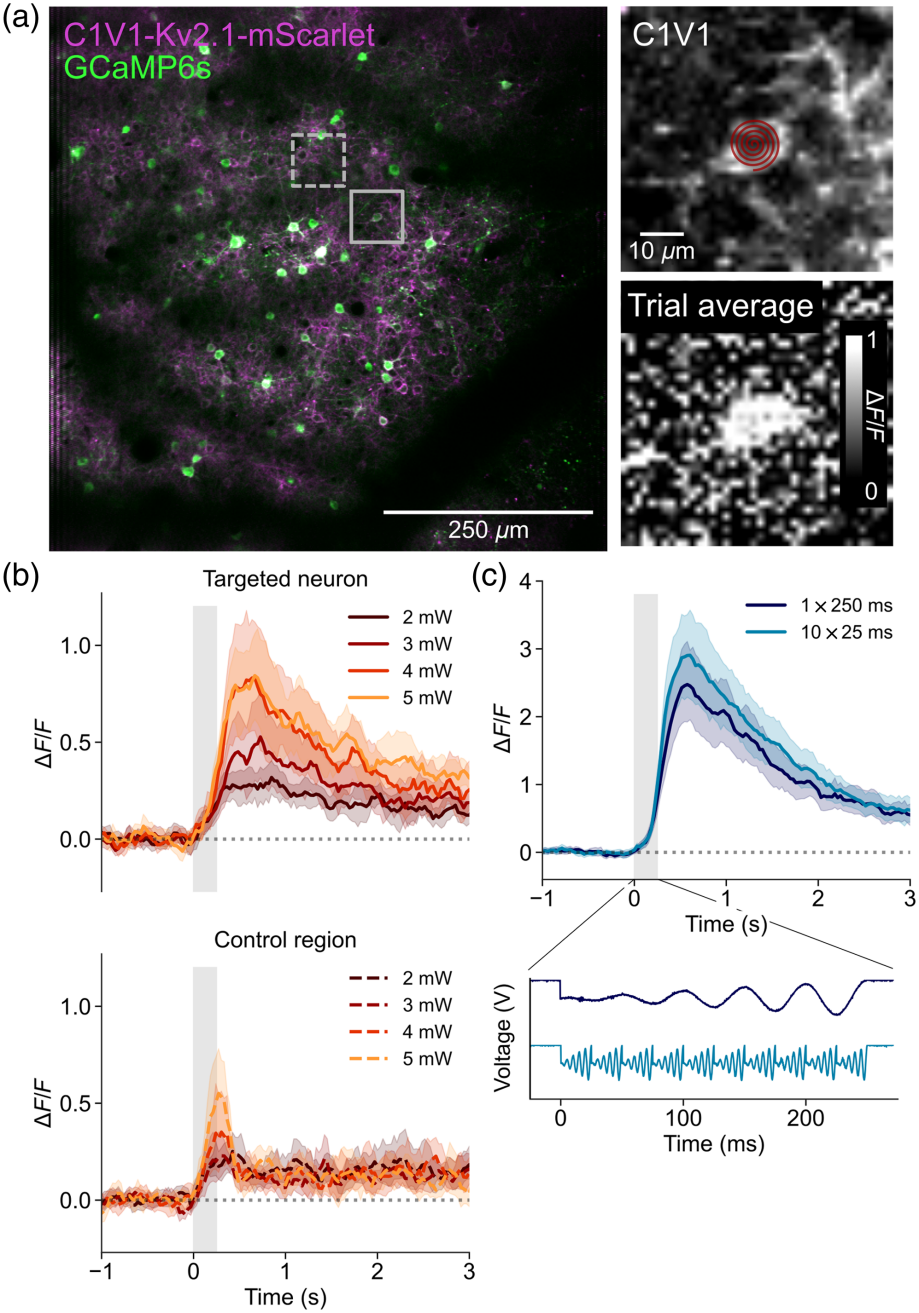
Optimizing photostimulation parameters for the detection of nonsaturated neuronal responses. (a) Left: example two-photon fluorescent image in layer 2 of the mouse somatosensory cortex. Neurons are labeled through viral expression with GCaMP6s and C1V1-Kv2.1-mScarlet (solid box, targeted neuron; dashed box, control region). Top-right: zoom of the targeted neuron showing the C1V1-Kv2.1-mScarlet labeling. An example spiral of 10  μm diameter is overlaid on the targeted neuron. Bottom-right: trial-averaged image of the targeted neuron in units of normalized change in fluorescence (ΔF/F) averaged across a period of 1.5 s post-photostimulation. (b) Top: mean ΔF/F traces ±95% CI for all neurons stimulated at each power level (different neurons for each power level; 2 mW, 3 mW, n=10 neurons each; 4 mW, 5 mW, n=9 neurons each). Bottom: mean ΔF/F traces ±95% CI for all control regions (dashed box in the left panel of (a)). (c) Mean ΔF/F traces ±95% CI for all neurons in each spiral condition (every cell was tested with both spiral conditions, n=24 cells). Bottom: voltage traces from the x galvanometric mirror that spirals the photostimulation beam showing the single-slow spiral (1×250  ms) and the fast repeated spirals (10×25  ms). The response post-photostimulation was not significantly different (independent t-test for 1×250  ms versus 10×25  ms: t=−1.06, p=0.293).

We needed to detect small changes in the neuron’s response to photostimulation to produce a curve of neuronal response versus the z offset of the photostimulation beam. To do this, we optimized the average power and frequency of photostimulation to prevent saturation of the neuronal response (as measured using calcium indicators). In this study, we used the normalized change in fluorescence (ΔF/F) of a common calcium indicator, GCaMP6s, as a proxy for neuronal response. We expressed GCaMP6s in neurons in the somatosensory cortex of mice alongside a perisomatic red-shifted opsin, C1V1-Kv2.1, conjugated to the fluorescent protein mScarlet in pyramidal cells only [Fig. 2(a)]. We identified single mScarlet-positive pyramidal neurons in the field of view to target for photostimulation.

First, we wanted to determine if the opsin was more effectively activated by repeatedly scanning the neuron with 10 fast spirals (25 ms) or a single slow (250 ms) spiral. For each neuron, we spiraled the photostimulation beam over the cell without any z offset while simultaneously imaging the GCaMP6s response at 920 nm. We stimulated the same neurons with fast and slow spiral scans, alternating which stimulation was tested first for each neuron. There was no significant difference in calcium response between slow spiraling and multiple fast spirals [Fig. 2(c)]. However, we did this test at 6 mW average power, which was potentially already a saturating power. We decided to use slow spirals (1×250  ms) for the experiments as this produced a slightly lower response (nonsignificant; independent t-test for 1×250  ms versus 10×25  ms: t=−1.06, p=0.293).

We adjusted the average power of the photostimulation beam in 1 mW steps from 2 to 5 mW as we noticed that 6 mW was resulting in very large, possibly saturating responses (>3 ΔF/F). Photostimulation of each neuron was repeated for three trials at each power level. The results showed that 4 and 5 mW average power of the photostimulation beam achieved similar responses, potentially indicating that the neurons had reached a maximal response at these powers [Fig. 2(b)]. We chose 3 mW average power for all further photostimulation experiments as the responses at 2 mW were too close to the noise floor estimated from nonphotostimulated control regions (100  μm from the photostimulation beam; see Sec. 2).

In summary, we chose a photostimulation laser power that produced submaximal responses at the central photostimulation plane (z=0  μm) to ensure that we could accurately sample the curve of neuronal GCaMP6s response at different photostimulation z offsets.

### 3.3 Measuring the Effect of OPSF Size on Neuronal Photostimulation Precision

**Fig. 3:**
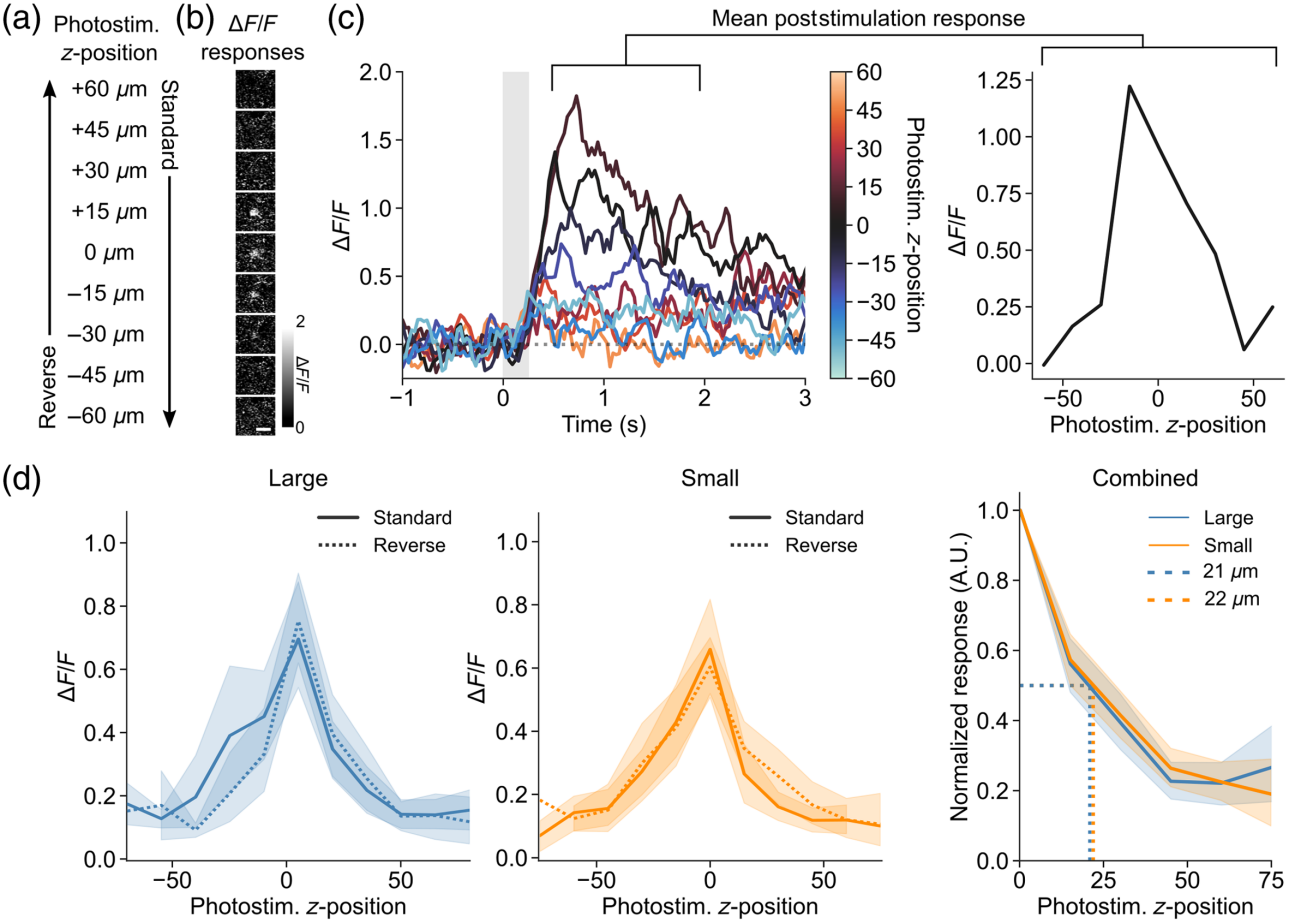
Accuracy of neuronal photostimulation in mouse somatosensory cortex layer 2 is not affected by variations in commonly used OPSF sizes. (a) Schematic of the different directions of photostimulation z travel. The photostimulation OPSF was stepped sequentially through the z dimension from top-to-bottom (+60 to −60  μm in 15  μm steps; “standard”) or bottom-to-top (“reverse”) by changing the hologram produced by the SLM. Three trials were repeated at each z position before moving to the next one. (b) Example mean ΔF/F images of post-photostimulation responses (mean of 1.5 s after stimulation end) for a targeted neuron that was photostimulated at each z position while being imaged at a fixed imaging plane (z=0  μm). Scale bar=20  μm. (c) Left: ΔF/F traces from a targeted neuron showing responses at each photostimulation z position. Right: the ΔF/F values in the period 1.5 s post-photostimulation were averaged to produce a neuronal response curve for axial photostimulation precision. (d) Left: curves showing mean post-photostimulation responses ±95% CI at each photostimulation z position for the large OPSF, for the two different directions (different cells for each direction; reverse, n=7 cells; standard, n=14 cells). The standard and reverse conditions were not statistically different (independent t-test for standard versus reverse at each z position: p>0.05). Middle: curves from the small OPSF (reverse, n=11 cells; standard, n=14 cells). Right: mean normalized photostimulation response ±95% CI versus photostimulation z position, a summary of the previous four curves for the large and small OPSFs. The half-width at half-maximum is indicated (dashed line) and not different for the two curves (independent t-test for large versus small OPSFs at each z position: p>0.05).

We wanted to test whether the axial spread of the OPSF affected the precision with which neurons can be photostimulated *in vivo*. To do this, we stimulated single neurons using different photostimulation z offsets with two different OPSFs. We again used mice expressing GCaMP6s and C1V1-Kv2.1 in neurons of the somatosensory cortex [Fig. 2(a)]. We varied the z offset of the photostimulation OPSF while simultaneously imaging the GCaMP response from the targeted neuron at z=0  μm, repeating three trials at each photostimulation z position. To account for any effects of opsin desensitization due to repeated exposure, the photostimulation z offset was either sequentially moved from +60 to −60  μm (“standard” direction) or from −60 to +60  μm [“reverse” direction; Fig. 3(a)]. Each neuron was only tested for a single OPSF size and a single direction (standard or reverse). This was done to ensure that as many different neurons were sampled as possible and to prevent as far as possible the potential for photodamage caused by excessive repeated trials. The resultant response from each neuron was recorded and the normalized change in fluorescence (ΔF/F) was measured post-stimulation (n=8 cells for each direction for small OPSF, n=7 cells for reverse, n=8 cells for standard for the large OPSF). The neuronal responses were highest at positions around z=0  μm and dropped to undetectable levels at the extreme z positions [Figs. 3(b) and 3(c)], implying a complete estimation of the entire response curve.

We compared the response curves from the standard and reverse directions of both OPSF sizes. Neurons that failed to respond with >0.35 ΔF/F in at least one z position were removed, so only responsive neurons were included (based on average responses from sample versus control regions; see Sec. 2.6). Additionally, due to the variation in the axial position of the imaging plane relative to the center of the targeted neuron soma, we aligned individual response curves by their maximum response [Fig. 3(d)]. The maximum offset of a curve was one z position (15  μm), which is about the size of a cell body and fits with the expected degree of uncertainty about the axial position when imaging neuronal cell bodies, that is, the identification of a cell’s axial position in the imaging plane was difficult to determine at a low zoom (required to avoid imaging-induced photostimulation; see Sec. 2.5), so the z=0  μm plane may have been at the very top or bottom of a cell. The standard and reverse directions were not significantly different from each other for either OPSF size (independent t test for standard versus reverse at each z position: p>0.05), so they were combined to improve the statistical power. Similarly, the two halves of the curve (above and below the cell) were combined; however, we do note that the shape of the curve above the neuron was steeper for both OPSF sizes. The FWHM of the photostimulation response curve for the large and small OPSFs was 44 and 42  μm, respectively [independent t-test for large versus small OPSFs at each z position: p>0.05; Fig. 3(e)].

**Fig. 4:**
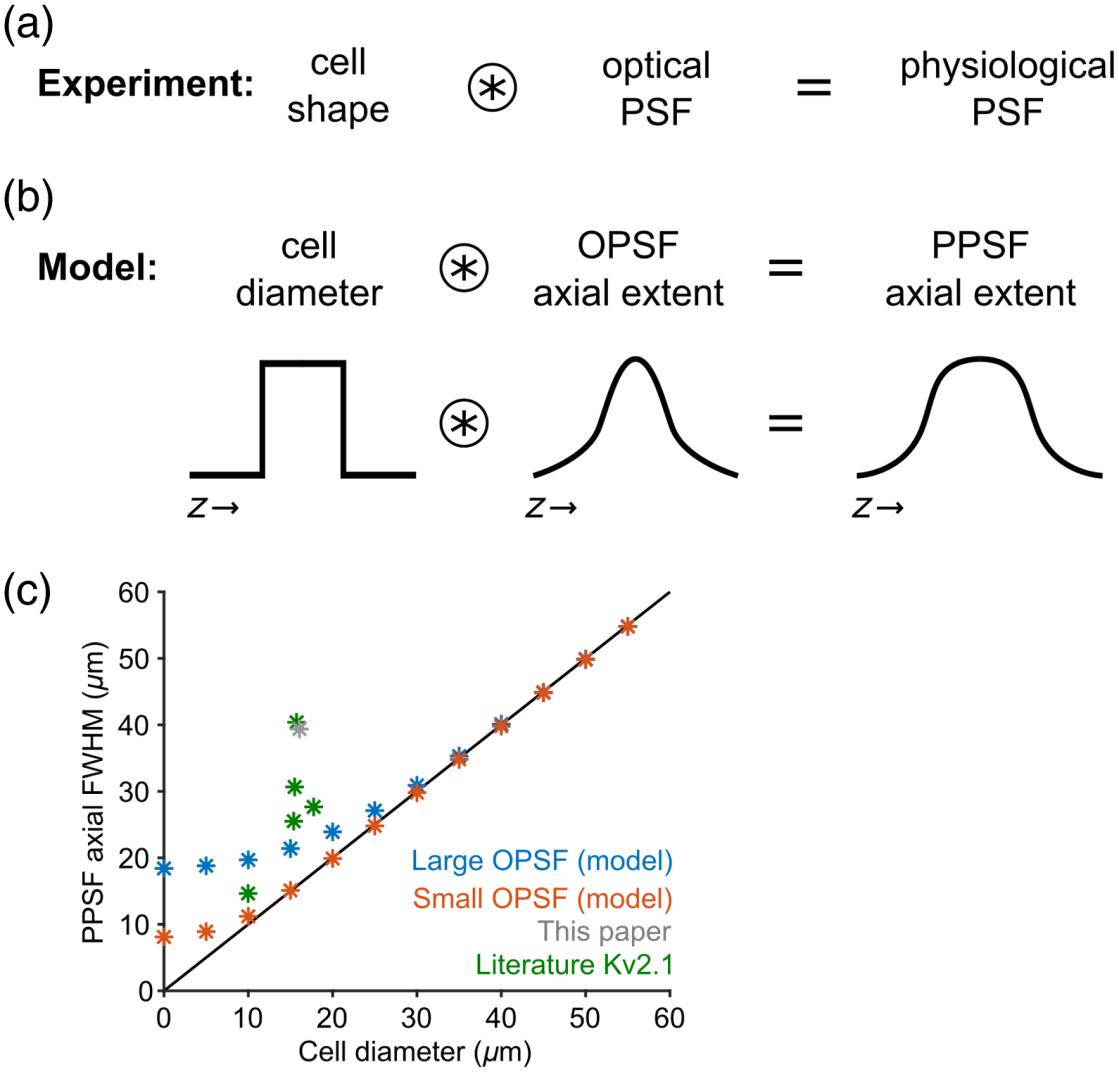
Modeling the physiological PSF for different cell shapes. (a) Hypothesis that the PPSF results from the convolution of the cell shape and the optical PSF shape. (b) The cell diameter was used to model the cell shape as a sphere, and the axial FWHM from the fluorescence signal of the OPSF was used to model the contribution of the OPSF. The cell diameter was modeled as a square wave, and the axial FWHM was a Gaussian. (c) A plot of the relationship between PPSF and cell diameter for the large (blue stars) and small (orange stars) OPSFs. Experimental results from this study (gray) and other literature using Kv2.1 (green) are included. The line of equality is also plotted (solid black line).

Our results show that the PPSF does not depend on the axial extent of the OPSF for the OPSFs that we measured and the biological system that we utilized (pyramidal cells in layer 2 of mouse neocortex). To further understand this result, we modeled the contribution of the cell size to the axial extent of the PPSF (Fig. 4). We used the width of a square pulse to model the cell diameter and convolved it with the axial extent of the large and small OPSFs to give the PPSF [Fig. 4(b)]. The results show that, as the cell diameter increases beyond the size of the OPSF axial FWHM, the PPSF axial FWHM begins to depend solely on the cell diameter [Fig. 4(c)]. Beyond a certain size (∼30  μm), both OPSF sizes converge on the same PPSF, breaking the dependence of PPSF on OPSF.

## 4 Discussion

We found that the axial precision of two-photon optogenetic excitation of pyramidal cells *in vivo* in mouse neocortex was not significantly affected when using OPSFs differing by more than a factor of two, from 8 to 19  μm axial FWHM. It can be challenging and expensive to achieve a tight photostimulation OPSF using an SLM; the results presented here indicate that putting effort into improving OPSF beyond a certain limit does not increase the accuracy of the PPSF. This was further confirmed by modeling the PPSF as a convolution of the axial extent of the OPSF and the cell diameter, showing that the PPSF converges for large cells when using the different OPSFs from this study. These results may be partly specific to the cell type, opsin, and light targeting methodology used here, although some principles described further in this section can be generalized.

Different types of cells, e.g., neurons from different species and/or different brain areas with different shapes and sizes, will require different photostimulation parameters. Variation in the expression of opsin and GCaMP also likely add variability to our measurements and could make it challenging to compare our study with other studies using different expression strategies. Additionally, our results may be affected by saturation, as the laser power often employed for two-photon excitation of opsins can be “saturating” in two related ways. There may be greater photon flux per excitation volume than required to excite all of the opsin molecules in that volume (i.e., saturating light dosage per excitation volume) or excite all of the opsin molecules in the somatic and nearby membranes (e.g., saturating activation of the opsin population per cell). Some kind of saturation seems likely given that the two-photon absorption cross section of channelrhodopsin-2 is high, which is likely the case for other opsins as well. These facts further imply that there is effective out-of-focus excitation, as shown for isolated cells^3^. Out-of-focus excitation further sacrifices resolution beyond the minimum set by the physics alone (i.e., the OPSF convolved with the cell).

Regarding the modeling of cell size versus PPSF, the model results are not meant to be an exact physical model of the entire optical and biological setting. Rather, the model is meant to illustrate the core effect that we think drives the results shown here. As such, the model results [Fig. 4(c)] do not match the experimental result [Fig. 3(e)] precisely in terms of absolute numbers. We think the discrepancy between the results and the model are most likely due to either opsin localization (perisomatic rather than somatic) or out-of-focus excitation. However, a complete physical model exploring these effects in quantitative detail is outside the scope of this work. The estimation of cell shape in our model could be adjusted to include the perisomatic dendritic compartments, as the Kv2.1 targeting sequence used for opsin expression in this study shows a perisomatic localization rather than a purely somatic localization. Additionally, the opsin is found only in the membrane and not across the entire cell volume; therefore, a better model of the cell shape could encompass the varying amount of opsin across the cell. A larger surface area at the middle of an approximately spherical cell may contain more opsin molecules than at the poles.

Regarding technical limitations, there is most likely an effect on OPSF when the SLM is imposing a z offset to the focus of the beam. We did not manage to measure this in this paper as we did not have the tools available. If we were to repeat this, we would use an electrically tuneable lens or other remote focusing device to measure the OPSF accurately at each z offset position.

Future work developing stronger opsins may help if they achieve their “strength” via increasing the conductance per protein, although this may also increase imaging-induced photostimulation in all-optical settings. However, simply increasing the expression level globally would not help because then the increased photocurrent from somatic activation will scale in the same way that increased photocurrent due to out-of-focus excitation of perisomatic regions increases. Truly somatic restriction instead of just perisomatic enhancement would be most beneficial. Crucially, further optimization of the spatial resolution at the optical level will not be beneficial (although see the limitations above).

In summary, the way forward to achieve optimal resolution may be to highly express (within healthy limits) high conductance opsin proteins in the somatic membrane alone. High expression and high conductance are desired to achieve sufficient depolarization to reach action potential threshold without needing to activate all of the opsin molecules and therefore avoid saturation and the concomitant out-of-focus excitation. However, care must be taken to avoid imaging-induced photostimulation with high conductance opsins. Finally, the light would need to be tailored to approximate a delta function (infinite at the center and zero everywhere else) in the axial direction as much as possible, at least such that the distribution of light is confined to a slab much thinner than the diameter of the target cells.

## Data Availability

Data can be freely accessed through GitHub at the following link, including all of the code and data to reproduce the figures in the paper: https://github.com/Packer-Lab/OPSF_vs_PPSF The raw data from calcium imaging is large (1 to 2 terabytes) and is freely available upon request.
